# Brugada Syndrome and Exercise: Is It Time for a Paradigm Change?

**DOI:** 10.3390/jcdd12030094

**Published:** 2025-03-06

**Authors:** Carolina Miguel Gonçalves, Adriana Vazão, Mariana Carvalho, Margarida Cabral, André Martins, Mónica Amado, Joana Pereira, Fátima Saraiva, Hélia Martins, Hélder Dores

**Affiliations:** 1 Unidade Local de Saúde da Região de Leiria, E.P.E., 2410-197 Leiria, Portugalmmcarvalho93@gmail.com (M.C.);; 2 Palliative Care Team, Internal Medicine, Emergency and Intensive Care Department, Hospital da Luz, 1500-650 Lisbon, Portugal; 3 NOVA Medical School, NOVA University Lisbon, 1600-560 Lisbon, Portugal; 4 CHRC-Comprehensive Health Research Center, 1099-085 Lisbon, Portugal; 5 Associate Laboratory REAL (LA-REAL), 1099-085 Lisbon, Portugal

**Keywords:** Brugada syndrome, exercise, sports, athletes, sudden cardiac death, implantable cardioverter defibrillator

## Abstract

Background: Despite the multiple benefits of exercise for health, exercise in the presence of arrhythmic disorders can trigger adverse clinical events, including sudden cardiac death (SCD). The aim of this narrative review is to summarize the most recent recommendations regarding physical activity and exercise in individuals with Brugada Syndrome (BrS). Methods: An advanced literature search was performed on the Pubmed and clinicaltrials.gov databases and published articles/clinical trials registered until September 2024 were analyzed. The final analysis included 33 articles. Results: Despite initial reports suggesting a higher risk of SCD in BrS, the risk is not as high as expected, and there is no evidence that exercise is an independent predictor. Therefore, scientific recommendations have become less restrictive. However, consensus on risk scores is lacking, making the evaluation of BrS a real challenge. The most recent recommendations emphasize individual evaluation, risk stratification, shared decision-making, and general preventive measures, allowing asymptomatic BrS patients as well as genotype positive/phenotype negative patients to participate in competitive sports, excluding sports under extreme conditions. Regarding patients with an implantable cardioverter defibrillator, both leisure and competitive sports may be considered in asymptomatic patients, avoiding contact sports. Conclusions: Research on the relationship between exercise and cardiovascular disease is evolving, but evidence-based recommendations for sports in BrS patients are scarce and further studies are needed.

## 1. Introduction

Physical activity and regular exercise training have several well-known benefits for health, including the prevention of primary and secondary cardiovascular disease (CVD) [1]. Despite these established advantages, some CVDs may carry an increased risk of sudden cardiac death (SCD), particularly in athletes [1]. In this setting, the main objective of preparticipation screening is the early diagnosis of conditions associated with a higher risk of SCD in this population [2].

In young athletes, the most frequent causes of SCD are inherited heart diseases, mainly cardiomyopathies and primary arrhythmias [3]. Recent registries have shown a high prevalence of sudden arrhythmic death, reported in up to 44% of post-mortem examinations [2]. Among the disorders responsible for these cases are cardiac channelopathies, particularly long QT syndrome and Brugada syndrome (BrS) [3,4].

The diagnosis of CVD in athletes raises several questions regarding sports eligibility. Classically, the decision often involves imposing restrictions or disqualification from competitive sports. However, despite the limited evidence, recent data demonstrate the possibility and benefits of continuing competitive sports in the presence of CVD, which is reflected in the recommendations of several scientific societies [2,5]. In fact, in different clinical scenarios, the association between exercise and SCD remains unclear, while the benefits of regular exercise are unquestionable [1,2]. The focus should not be exclusively on the diagnosis of the disease, but rather on evaluating the risk for SCD and other clinical outcomes related to exercise training in specific clinical settings.

While restrictions should be implemented in the presence of high-risk features, such as conditions where exercise could trigger complications or worsen the disease, the recommendations can be more liberal in low-risk individuals. BrS is an example of a condition with a broad spectrum of risk and where the association between SCD and exercise remains controversial [5,6,7,8].

The aim of this review is to summarize the more recent recommendations regarding physical activity and exercise training in individuals with BrS.

## 2. Methods

An advanced literature search on PubMed and other research databases was performed using the following mandatory keywords, “Brugada Syndrome”, “Sports”, “Exercise”, “Athletes”, “Physical Activity”, and “Physical Exertion”, resulting in a total of 389 articles. Furthermore, a search for clinical trials was conducted (http://clinicaltrials.gov, accessed on 1 September 2024).

Papers published until September 2024 were analyzed, and 30 articles, including 11 society recommendations/guidelines, 10 original articles, 6 reviews papers, 2 clinical trials, and 1 letter to the editor, were included in this narrative review. All of the excluded articles did not meet the purpose of this review. Three other articles of interest were included.

## 3. Results

### 3.1. Definition and Diagnosis

BrS is a primary electrical disease that gained attention within the scientific community in 1992 with several publications by Dr. Josep Brugada and Dr. Pedro Brugada [1,7,9]. Its prevalence ranges from 0.02 to 0.1% in Europe and is apparently higher in Southeast Asia, although this is not reported in the most recent recommendations [4]. Furthermore, BrS is an autosomal-dominant disease mostly associated with the SCN5A gene, leading to changes in sodium currents and disturbances in the cardiac action potential [4,7]. Although genetic testing for SCN5A is advised, its diagnostic yield is only 20% to 30%. Additionally, despite recent published data, its prognostic significance remains controversial [3,7]. Other mutations were found (GPD1L42, SCN1B, CACNA1C or CACNB2b), but they are very uncommon [4,9].

According to the most recent European recommendations, BrS is diagnosed in patients with a spontaneous type 1 pattern (>2 mV J point elevation with coved ST elevation and T-wave inversion in ≥1 right precordial lead) in an electrocardiogram (ECG) without other heart disease (recommendation I C). In the case of an induced type 1 pattern (by drugs or fever), other features are needed for the diagnosis: cardiac arrest due to ventricular fibrillation or polymorphic ventricular tachycardia (recommendation I C), arrhythmic syncope, nocturnal agonal respiration, and family history of BrS or SCD in relatives younger than 45 years old (recommendation IIa C). Nevertheless, these recommendations include less evidence for isolated induced type 1 ECG patterns (recommendation IIb C) [3].

**Figure 1:**
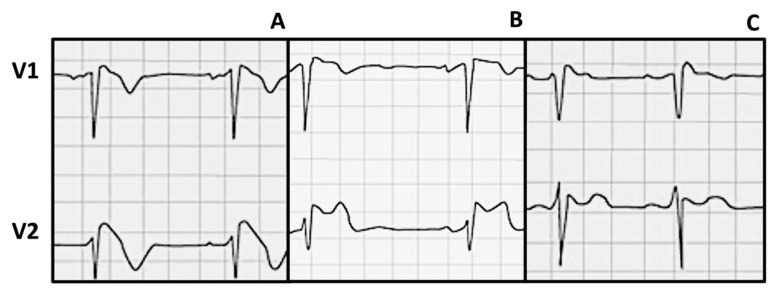
Brugada ECG patterns (A: type 1; B: type 2; C: type 3).

Other Brugada ECG patterns have been described (Figure 1). Type 2 is characterized by at least 2 mm of J point elevation and 1 mm ST elevation with a positive/biphasic T wave, while type 3 has less than 2 mm and 1 mm elevations, respectively [7]. In cases where no unequivocal spontaneous type I ECG pattern exists and BrS is suspected, a sodium channel blocker test is recommended, and no other considerations are made regarding type 2 and 3 in the current recommendations [3].

The Brugada pattern may be challenging to diagnose due to its fluctuation throughout the day, influenced by the autonomic tone. It often becomes more evident during periods of increased vagal tone—in the early evening and in afternoon [9,10]. ECG with leads V1 to V3 placed at a higher intercostal space has a higher sensitivity for detecting the pattern.

**Figure 2:**
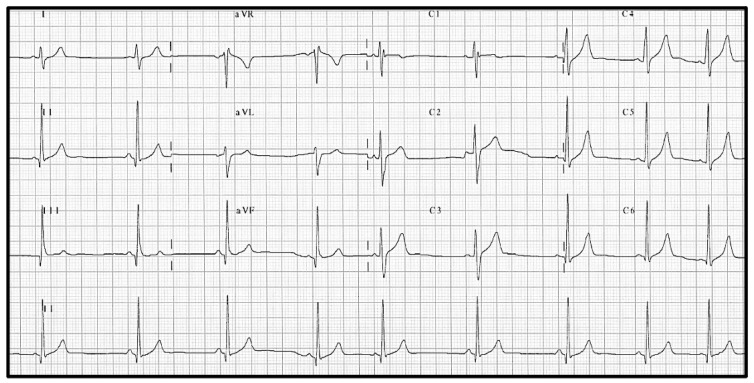
ECG with an incomplete right bundle branch block in an athlete.

Specifically in athletes, the distinction between the Brugada pattern and an incomplete right bundle branch block (IRBBB) (Figure 2), a common physiological ECG finding in this population, may be challenging [11]. Although Corrado et al. characterize IRBBB as an R’ wave in leads V1 and V2 associated with S wave in leads I and V6, and without ST segment elevation in the right precordial leads, they acknowledge the possibility of a provocative test for difficult cases [12]. These authors also propose using the ratio between the maximal ST segment amplitude measured at the J point (ST_J_) and after 80 milliseconds (ST_80_) and the QRS interval duration to help make a differential diagnosis with benign early repolarization pattern. A ST_J_/ST_80_ ratio lower or equal to 1 had a sensitivity of 87% and a specificity of 100% for identifying the athletes without BrS [11].

Furthermore, several groups have investigated beta angle measurements to help in this differential diagnosis [13,14]. Chevallier et al. proposed a predictive model for the response to provocation test using beta and alpha angles in ECG, with an optimal cutoff of more than 50° and 58° for the alpha and beta angles, respectively, the latter being more sensitive and specific [14].

### 3.2. Risk Stratification and Mechanisms for SCD

Risk stratification in BrS patients is challenging and remains under investigation [3,4,7,15]. A 0.5% risk of arrhythmic events per year is reported in asymptomatic BrS patients, which constitute most of the patients, while those with unexplained syncope have a four-fold increased risk [3]. Patient background, characterization of symptoms, mainly syncope episodes and its triggers, ECG markers (Table 1), such as QRS fragmentation, the early repolarization pattern (ERP), the Brugada pattern in inferior or lateral leads, and data from electrophysiological studies (EPS) are suggested to improve this risk stratification [3,10]. However, no specific risk scores are recommended [3]. Whether an aggravated Brugada pattern during exercise or early recovery on exercise testing or EPS identifies a higher risk for SCD is unproven [2] and the prognostic significance of a positive EPS also remains controversial [7,15]. The concept of the “Brugada burden”—the more extensive the ECG alterations in space (peripheral and precordial leads) and in time (persistence of electrocardiographic findings), the greater the risk of arrhythmic events—is emerging in the scientific community [10,16,17].

SCD in BrS patients usually occurs at rest [18], and arrhythmias are not adrenergic-induced [5]. It is hypothesized that SCD in these patients may be caused by ventricular arrhythmias during exercise, triggered by conduction defects and increased vagal tone, inherent to athletic conditioning [2,4,18]. However, the evidence is scarce and there are no studies validating this hypothesis [2,5,19]. A small study in non-athletes conducted by Chanavirut et al. showed higher parasympathetic activation during the recovery phase in exercise testing evaluated by heart rate variability [20]. In line with parasympathetic activation in the recovery phase, a retrospective observational study suggested an association between premature ventricular contractions (PVCs), which were observed in parallel with ST segment augmentation, and future occurrence of ventricular fibrillation. Nevertheless, the authors conclude that exercise tests are safe in these patients [8].

Hyperthermia during strenuous and endurance exercise may also be associated with arrhythmic events in BrS patients [4,5,18].

### 3.3. Recommendations for Physical Activity and Exercise in BrS

**Figure 3:**
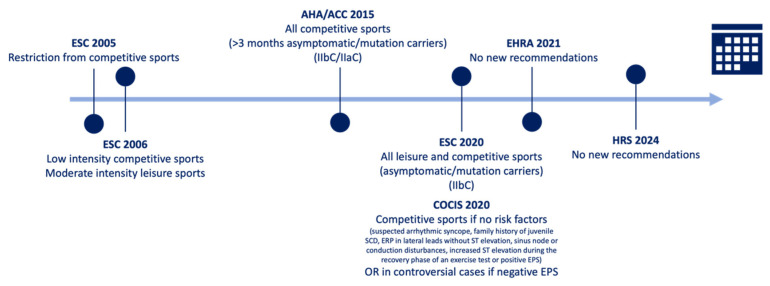
Evolution of the recommendations regarding exercise in BrS patients (EPS: electrophysiological study; ERP: early repolarization pattern; SCD: sudden cardiac death).

There is a general gap in the evidence on sports cardiology, particularly regarding the lack of prospective randomized clinical trials [21]. Recommendations about physical activity and exercise training in BrS patients are scarce, with disqualification from competitive sports being the traditional clinical decision [4,7,22,23]. Nevertheless, as no robust evidence shows an increased risk of SCD with exercise, total exercise restriction in BrS is not advocated [5,6,7,8]. With several studies reporting a low risk of SCD during exercise in individuals with BrS, the recommendations have become more liberal [7] (Figure 3 and Table 2).

Preventive measures, such as avoiding hyperthermia, saunas, steam rooms, hot tubes, electrolytic disturbances, dehydration, heavy meals, alcohol, prolonged endurance sports, or exercise in warm and humid environments, are generally recommended [2,5,18,24].

According to the 2006 ESC recommendations, only low-intensity competitive sports and moderate-intensity leisure sports were allowed in spontaneous or induced BrS patients (with symptoms or inducible), while in genotype-positive/phenotype-negative patients, low-to-moderate intensity competitive sports were permitted. There was a relative contraindication for sports with intrinsic risk due to potential complications from syncope episodes, such as trauma [18]. In contrast, the 2015 American recommendations suggested that competitive sports may be considered in asymptomatic (more than 3 months) BrS athletes (recommendation IIb C), while all intensities were allowed for genotype-positive/phenotype-negative patients (recommendation IIa C), with general preventive recommendations [24].

The most recent 2020 European recommendations consider both leisure and competitive sports for asymptomatic BrS patients or those with only an inducible pattern, as well as for genotype-positive/phenotype-negative patients. However, activities associated with an increased core temperature (above 39 °C), such as endurance sports under extreme conditions, are excluded (recommendation IIb C) [2].

Furthermore, the 2020 Italian cardiological recommendations (COCIS) are more specific, considering competitive sports in spontaneous BrS patients without any potential risk factors, namely suspected arrhythmic syncope, family history of SCD (under 40–45 years), ERP in lateral leads without ST elevation, sinus node or conduction disturbances (e.g., first-degree atrioventricular block), increased ST elevation during the recovery phase of an exercise test, or positive EPS. Drug-induced type 1 ECG patterns without risk factors, controversial BrS cases with negative EPS, and type 2 or type 3 Brugada patterns without risk factors are also eligible [15].

Similar preventive recommendations were outlined in the 2021 European Heart Rhythm Association (EHRA) position paper, which advised measuring body temperature during training sessions [19], and reiterated in the recently published 2024 HRS (Heart Rhythm Society) expert consensus [5]. As highlighted in other fields of sports cardiology, the role of the shared decision-making processes is emphasized, involving the athlete, their families, exercise-related staff, cardiologists, other physicians, and healthcare professionals [21].

### 3.4. Recommendations for Physical Activity and Exercise in BrS with ICD

**Figure 4:**
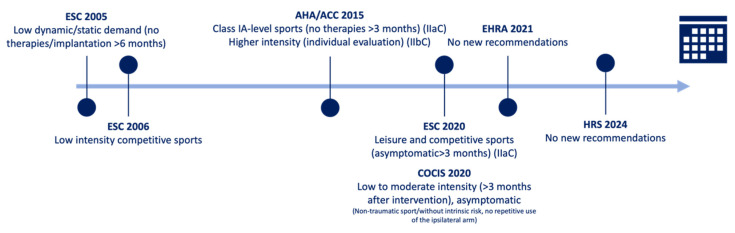
Evolution of the recommendations regarding exercise in BrS patients with ICD.

Recommendations for the eligibility of individuals with an implantable cardioverter defibrillator (ICD) to participate in sports have also changed over time (Figure 4 and Table 3). In line with the 2005 ESC recommendations and the 2006 European document, ICD implantation was a reason for disqualification from competitive sports, except in cases of low-intensity sports (e.g., golf, bowling) and in low-to-moderate intensity leisure sports [18,23,24]. In these cases, sports participation was only allowed six months after implantation or in the absence of symptoms [23].

Similarly, the 2015 American recommendations for competitive athletes with BrS allowed class IA-level sports (low static and dynamic component) if athletes were free from device therapies for more than three months (recommendation IIa C), but considered the possibility of higher intensity sports after an individualized evaluation (recommendation IIb C) [25].

On the other hand, the 2020 ESC Guidelines on sports cardiology, consider both leisure and competitive sports in BrS patients who have been asymptomatic for more than three months (recommendation IIa C), advising against contact sports [2,19]. In fact, these recommendations highlight that sports recommendations should be based on the underlying disease (recommendation IB) and that shared decision-making process should be considered (recommendation IIa C) [2]. No new recommendations were given in the subsequent 2021 EHRA position paper [19].

The 2020 COCIS recommendations consider patients to be eligible for low-to-moderate intensity sports three months after device implantation/intervention with the device, in asymptomatic individuals, and in those without prohibitive heart disease. Non-traumatic sports and activities that do not frequently use the ipsilateral arm are preferred, but these recommendations are not specific to BrS patients [15]. Regarding the type of ICD and according to the 2024 HRS recommendations, subcutaneous devices may have advantages in sports with repetitive arm use, while they may be a disadvantage in contact sports [5]. The choice between the different devices is beyond the scope of this review.

Similarly, several surveys and international registries have addressed the participation of pediatric patients with ICDs in sports, and the 2021 PACES (Pediatric and Congenital Electrophysiology Society) consensus recommends shared a decision-making process based on the underlying disease and risk [26], without individualizing treatment to BrS.

Although several studies do not report a higher incidence of device malfunction with sports participation, the 2024 HRS recommendations highlight that safety studies in sports are lacking, particularly for devices other than endovascular (e.g., subcutaneous, extravascular or epicardial). Rates of lead malfunction in collision sports are not available, although direct trauma, clavicular crush, sudden deceleration, weightlifting, and hyperextension injuries may result in device malfunction [5]. Nevertheless, to prevent lead dislodgement, avoiding lifting for four to six weeks after the implantation of a transvenous device or two weeks after generator replacement is the standard approach [5].

Appropriate and inappropriate shocks have been observed during physical activity, with no difference in their frequency between training and ordinary physical activity [5]. Device optimization is necessary to reduce inappropriate shocks. While the COCIS guidelines recommend programming the device to at least 20 beats per minute above the maximal heart rate during physical exertion [15], the American guidelines suggest long duration and high-rate cutoff programming, individually adapted. Maximal exercise testing and Holter monitors are crucial to optimal device programming [5,15]. Before returning to exercise and after each round of therapy, leads and system revision, device optimization in inappropriate therapies, and management of appropriate therapies are needed [5]. This evaluation implies a comprehensive and multidisciplinary approach.

**Figure 5:**
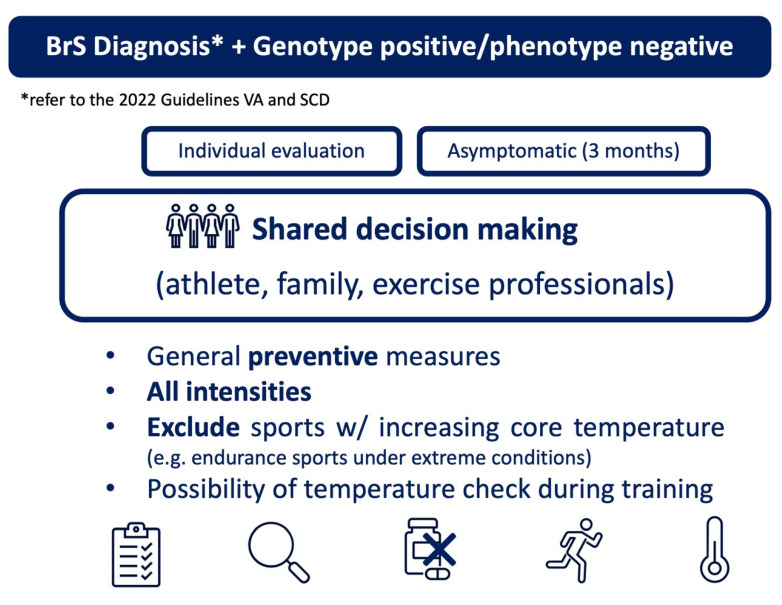
Proposed pathway for physical activity and exercise in BrS patients.

**Figure 6:**
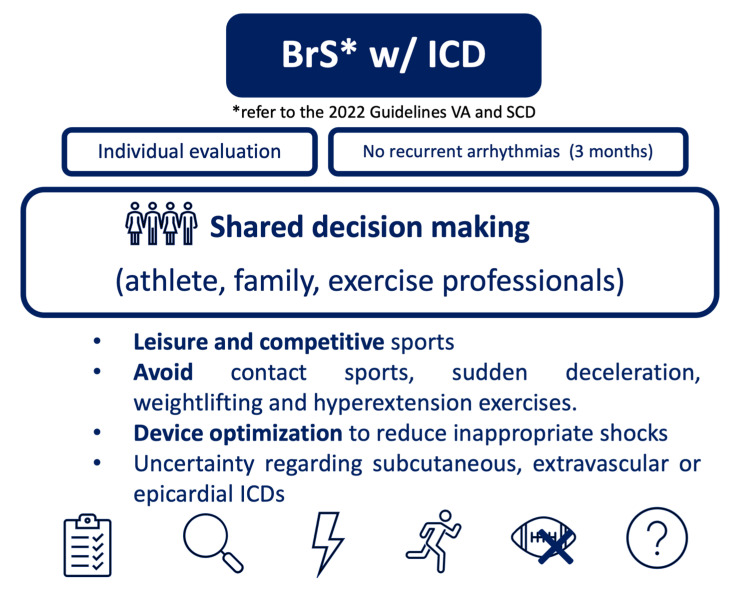
Proposed pathway for physical activity and exercise in BrS patients with ICD.

As the knowledge about this disease is constantly evolving, two clinical pathways have been proposed, in alignment with the most recent publications (Figure 5 and Figure 6).

### 3.5. Studies Involving Exercise in BrS Patients

There are several studies involving exercise in BrS patients (Table 4), mostly including exercise testing, but none specifically performed in athletes.

Regarding the diagnosis, Pichara et al. proposed using high precordial leads in the treadmill exercise testing protocols, with passive recovery in the supine position to increase diagnostic yield [10]. Pospiech et al. used cycle ergometer exercise testing to study ECG alterations (beta angle—measured between the upslope of the S wave and the downslope of the r’ wave) to discriminate between BrS patients [13].

In terms of risk stratification, several groups have also studied exercise testing. Morita et al. showed an association between PVC during the early recovery phase of treadmill exercise testing (1.5 to 3 min) and the future occurrence of ventricular fibrillation. These PVCs occurred in parallel with ST augmentation, and the authors suggest parasympathetic rebound as the mechanism for both findings. However, this was a retrospective observational study, with no control group, a short follow-up period with a small number of cardiac events, and a scarcity of evaluations of other ECG markers, limiting the generalizability of the results [8].

Romero at al. proposed novel depolarization indices during cycle ergometer exercise testing that may help to improve risk stratification in asymptomatic BrS patients [29]. Subramanian et al. related S wave upslope duration at peak exercise, J point elevation in aVR more than 2 mm during late recovery, and delayed heart rate recovery with future arrhythmic events [30]. In line with these data, Calvo et al. used time–frequency heart rate variability analysis during exercise testing to propose a novel predictive model [31]. In 2010, Makimoto et al. found an association between ST segment augmentation and cardiac events, which also occurred with larger heart rate recovery in asymptomatic patients, suggesting a relationship with higher parasympathetic activity [32]. Additionally, Amin et al. reported several ECG findings during exercise testing in BrS patients compared to healthy subjects, but these were not associated with symptoms or positive EPS [33]. Nevertheless, prospective validation of these models is needed.

### 3.6. Studies Ongoing About Exercise in BrS

After a search for registered clinical trials that addressed BrS and exercise (at https://clinicaltrials.gov, accessed on 1 September 2024), only two trials were found [27,28]. These trials included small samples of patients with BrS and the evaluation of safety during exercise was not the primary purpose. In children (6 to 18 years old) with inherited arrhythmic disorders and cardiomyopathies, Boisson et al. (NCT04650009) reported good adherence to the 2020 European recommendations and aerobic fitness evaluated by cardiopulmonary exercise testing [27]. This was a pilot study for the QUALIMYORYTHM trial (NCT04712136), a multicenter observational study in children (6 to 17 years old), aiming to evaluate the quality of life, particularly according to the level of physical activity, using a fitness tracker [28]. To the authors’ knowledge, no results have been published to date regarding BrS.

## 4. Final Considerations and Conclusions

SCD risk stratification assumes a central role in BrS, but several points remain controversial and challenging for clinicians. Scientific recommendations regarding exercise in BrS patients are evolving, with the most recent guidelines being less restrictive and recommending a shared decision-making process. It is evident that research into sports cardiology remains scarce, and BrS is not an exception, as reported in the present review.

After careful individual evaluation, risk stratification, and the adoption of general preventive measures, competitive sports (excluding sports under extreme conditions) should be allowed for asymptomatic BrS. Nevertheless, Italian recommendations specify several risk factors that may lead to the restriction of competitive sports in asymptomatic patients.

On the other hand, competitive sports should be permitted in asymptomatic BrS patients (for more than 3 months) with an ICD according to the 2020 ESC guidelines, avoiding contact sports. Conversely, the Italian recommendations are more restrictive regarding the intensity of sports that ICD patients should be involved in, but no recommendations are specific to BrS patients.

In conclusion, exercise does not appear to significantly increase the risk of SCD in BrS patients, but further evidence is needed to clarify the clinical management and recommendations in this field. Randomized controlled trials with longer follow-up periods are needed to understand the impact of exercise on these patients.

## Figures and Tables

**Table 1:** SCD risk stratification.

ECG/Exercise Test Finding (Reference)	Implications
QRS fragmentation (ECG) [3]	Linked to higher SCD risk but low utility in intermediate-risk patients
Early repolarization pattern (ECG) [3]	Linked to higher SCD risk but low utility in intermediate-risk patients
Brugada pattern in inferior or lateral leads (ECG) [10]	Linked to higher SCD risk
Aggravated Brugada pattern during exercise/early recovery (exercise testing) [2]	Controversial
Brugada “burden” (several leads and persistence in time of the pattern) [10,16,17]	Linked to higher probability of arrhythimc events

**Table 2:** Scientific recommendations for exercise in BrS patients (when available class/level of evidence is presented).

Society/Year	Recommendations
ESC 2005 [23]	No competitive sports.
ESC 2006 [18]	Competitive sports with low cardiovascular demand. Leisure-time sports with moderate cardiovascular demand.
AHA/ACC 2015 [24]	All competitive sports (IIb C). Asymptomatic athlete with genotype-positive/phenotype-negative—all competitive sports with appropriate preventive measures (IIa C), including the following: Avoidance of drugs that exacerbate BrS, electrolyte/hydration replenishment, avoidance of hyperthermia, acquisition of a personal automatic external defibrillator, and establishment of emergency action plans. All competitive sports for an athlete previously symptomatic (asymptomatic for >3 months) or with evidence of an electrocardiographic BrS pattern (IIb C): Assuming appropriate preventive measures and treatments.
ESC 2020 [2]	Sports activities * not associated with an increase in core temperature > 39 °C (e.g., endurance events under extremely hot and/or humid conditions) (asymptomatic, mutation carriers, asymptomatic inducible ECG pattern) (IIb C). (* All sports, except endurance sports with increased core temperature.)
COCIS, Italian 2020 [15]	Competitive sports if no risk factors (suspected arrhythmic syncope, family history of SCD (age < 40–45 years), early repolarization pattern in lateral leads without ST elevation, sinus node or conduction disturbances, increased ST elevation during the recovery phase of an exercise test or positive EPS). Competitive sports in Brugada types 2 and 3 or a positive drug test and no risk factors (above-mentioned). Competitive sports in controversial cases if negative EPS.
EHRA 2021 [19]	All sports not associated with an increase in core temperature > 39 °C (e.g., endurance events under extremely hot and/or humid conditions)—(asymptomatic, mutation carriers, asymptomatic athletes with an inducible ECG pattern) (“may do”).
HRS 2024 [5]	No evidence to support exercise restriction: Aggressive treatment of fever (1 C-LD), avoidance of hyperthermia (1 C-EO), and triggers for arrhythmias (1 B-NR).

BrS: Brugada syndrome; ECG: electrocardiogram; EO: expert opinion: LD: limited data; EPS: electrophysiological study; NR: nonrandomized; SCD: sudden cardiac death.

**Table 3:** Scientific recommendations regarding exercise in BrS patients with ICD (when available class/level of evidence is presented).

Society/Year	Recommendations
ESC 2005 [23]	Low–moderate dynamic and low–static sports—except sports with risk of bodily collision (I A,B). At least 6 months after the implantation/arrhythmic episode with defibrillator intervention. Cut-off heart rate for the ICD needs to be set appropriately.
ESC 2006 [18]	No competitive sports—except with a low cardiovascular demand and no risks due to syncope (>6 weeks after implantation). Leisure-time sports allowed (>6 weeks after implantation). assessment of expected maximal sinus rate/atrial fibrillation, with prophylactic antiarrhythmic/bradycardic therapy. reconsideration of the 6-week avoidance of sports after ICD intervention. avoidance of bodily impact, extreme ipsilateral arm movements, strong magnetic fields. Relative contraindication for sports with risks to the patient or others
AHA/ACC 2015 [25]	Class IA-level sports (athletes free from device therapies >3 months) (IIa C) Higher intensity depending on individual evaluation (if free from device therapies >3 months) (IIb C): Considering the likelihood of appropriate/inappropriate shocks and the potential for device-related trauma in high-impact sports.
ESC 2020 [2]	Leisure and competitive sports (without recurrent arrhythmias >3 months after ICD implantation) (IIa C): Shared decision-making.
COCIS, Italian 2020 [15]	Low-to-moderate intensity (>3 months after therapies and in asymptomatic individuals). Sports with a lower risk of trauma and the avoidance of repetitive use of the ipsilateral arm (preferred).
EHRA 2021 [19]	Leisure and competitive sports (no recurrent event > 3 months) (“may do”): Shared decision-making.
HRS 2024 [5]	No specific recommendations.

**Table 4:** Studies involving exercise in BrS patients.

Author/Year	N	Conclusions
Boisson et al., 2021 [27]	32	Good compliance with current sports guidelines and good aerobic fitness in children with inherited arrhythmic disorders and cardiomyopathies.
Amedro et al. [28]	214	No overall results have been published.
Pichara et al., 2021 [10]	163	Treadmill exercise testing protocol (high precordial leads and passive recovery phase in the supine position) increased the diagnostic yield of BrS.
Morita et al., 2020 [8]	307	Premature ventricular contractions in the early recovery phase of treadmill exercise test were associated with future occurrence of ventricular fibrillation in BrS patients.
Romero et al., 2020 [29]	110	Depolarization dynamic analysis during cycle ergometer exercise testing may help identify higher risk asymptomatic BrS.
Subramanian et al., 2017 [30]	163	Increased S wave upslope duration ratio in the precordial leads (peak exercise), augmentation of J point elevation in lead aVR and delayed heart rate recovery (late recovery) were independent predictors of future major arrhythmic events in BrS.
Calvo et al., 2018 [31]	105	Proposed model using time-frequency heart rate variability analysis during cycle ergometer exercise testing to identify higher risk BrS patients.
Chanavirut et al., 2015 [20]	34	BrS patients had higher parasympathetic and lower sympathetic activation during the recovery period after cycle ergometer exercise testing.
Pospiech et al., 2015 [13]	63	ECG alterations (beta angle) during cycle ergometer exercise testing were more pronounced in BrS patients compared to healthy subjects.
Makimoto et al., 2010 [32]	195	ST elevation augmentation during early recovery (1 to 4 min) of the treadmill exercise test was associated with future cardiac events in asymptomatic BrS patients.
Amin et al., 2009 [33]	85	Several ECG alterations during exercise testing were reported in BrS patients (QRS widening in SCN5A positive BrS patients and QT prolongation in BrS patients) compared to healthy subjects.

## Data Availability

Not applicable.
